# Admission Leuko-Glycemic Index and In-Hospital Mortality in Patients with Acute Surgical Abdomen: An Exploratory Retrospective Cohort Study

**DOI:** 10.3390/medicina62101879

**Published:** 2026-09-28

**Authors:** Andrei Modiga, Vlad-Olimpiu Butiurca, Cristian Marius Boeriu, Teodora Sorana Truta, Emilia Turucz, Vasile-Bogdan Halațiu, Călin-Dragoș Molnar, Bogdan Macedon, Daniel Dragoș Stanca, Călin Molnar

**Affiliations:** 1 Doctoral School of Medicine and Pharmacy, George Emil Palade University of Medicine, Pharmacy, Sciences and Technology of Târgu Mureș, 540139 Târgu Mureș, Romania; andrei.modiga@umfst.ro; 2 Faculty of Medicine, George Emil Palade University of Medicine, Pharmacy, Sciences and Technology of Târgu Mureș, 540139 Târgu Mureș, Romania; vlad.butiurca@umfst.ro (V.-O.B.); sorana.truta@umfst.ro (T.S.T.); emilia.turucz@umfst.ro (E.T.); bogdan.halatiu@umfst.ro (V.-B.H.); calin.molnar@umfst.ro (C.M.); 3 Clinical Emergency Department (UCPU-SMURD), Clinical Emergency County Hospital, 540136 Târgu Mureș, Romania; 4 Clinical Department of Surgery, Clinical Emergency County Hospital, 540136 Târgu Mureș, Romania

**Keywords:** acute surgical abdomen, in-hospital mortality, inflammation-based ratios, leuko-glycemic index, neutrophil-to-lymphocyte ratio, risk stratification

## Abstract

*Background and Objectives*: The leuko-glycemic index (LGI) is an emerging immunometabolic biomarker that integrates systemic inflammation and stress hyperglycemia and has shown prognostic value in various acute conditions. Its utility in patients with acute surgical abdomen remains unknown. We hypothesized that elevated admission LGI values would be independently associated with higher in-hospital mortality in patients with acute surgical abdomen and would show discriminative performance at least comparable to established inflammation-based indices (NLR, MLR, PLR, SII). *Materials and Methods*: We conducted a single-center retrospective cohort study including 1927 adults admitted with acute surgical abdomen who underwent emergency surgery between March 2019 and March 2021. Patients were stratified according to in-hospital outcome into survivors (Group 1, *n* = 1625) and non-survivors (Group 2, *n* = 302). Demographic, hematological, and biochemical parameters, including inflammation-based ratios (NLR, MLR, PLR, SII) and the LGI, were collected upon admission. Non-parametric tests were used for between-group comparisons, and receiver operating characteristic (ROC) curve analysis assessed the discriminative performance of each index. Multivariable logistic regression was used to assess the independent association of LGI with in-hospital mortality. *Results*: Non-survivors were older and exhibited more pronounced hematologic and biochemical derangements, including higher leukocyte, neutrophil, glucose, urea, creatinine, and AST levels, along with lower hemoglobin, hematocrit, red blood cell, and lymphocyte counts. All inflammation-based indices were significantly elevated in Group 2 (all *p* < 0.0001). LGI was markedly higher in non-survivors (median 1986 [IQR 1263–3113] vs. 1568 [980–2083]; *p* < 0.0001) and showed the highest AUC among the indices tested (0.646, 95% CI 0.607–0.685), significantly higher than MLR, PLR and SII (DeLong *p* ≤ 0.0002) and nominally higher than NLR (*p* = 0.042). An LGI cut-off of 1665 yielded a sensitivity of 60.17% and a specificity of 57.73% for in-hospital mortality. In multivariable analysis, log-transformed LGI remained independently associated with in-hospital mortality (OR 1.44 per 1 SD, 95% CI 1.20–1.73; *p* = 0.0001). *Conclusions*: Admission LGI was independently associated with in-hospital mortality and outperformed the inflammation-based ratios tested, but its standalone discrimination was modest and its threshold unstable; these findings do not support its use for triage or risk stratification, and external prospective validation would be required before any clinical role could be considered.

## 1. Introduction

Acute abdominal pain is one of the most common reasons for emergency room presentation worldwide, with underlying causes ranging from harmless to acute life-threatening conditions, which may require immediate life-saving surgical intervention [1]. The broad array of underlying causes and clinical presentations poses a challenge for emergency physicians around the world, while only 20 to 25% of patients are diagnosed with an acute surgical abdomen, requiring hospitalization and surgical management [2,3].

The acute surgical abdomen is a surgical emergency, usually presenting as sharp, sudden, continuous, high-intensity abdominal pain, with a duration of less than 72 h, associated with one or more of the following symptoms: nausea, vomiting, fever, constipation, diarrhea, inappetence, abdominal distension and guarding, and jaundice, ultimately leading to shock and death [4,5]. To avoid misdiagnosis and delays in treatment, a combination of rigorous history-taking, an exhaustive clinical examination, specific and non-specific paraclinical examinations, and a broad differential diagnosis is needed [6,7].

Contingent upon the main pathology leading to acute abdomen and the patient’s age, mortality rates range vastly from 2 to 14%, with the highest incidence among the elderly [8]. Additionally, studies have identified a positive correlation between the presence of primary diseases, for instance, cardiovascular diseases, chronic kidney disease, active malignancies or current presentations with signs of shock, systemic inflammatory response syndrome (SIRS) and multi-system organ failure (MSOF) and an augmentation in mortality rates [9]. In regard to the overall outcome of patients diagnosed with acute surgical abdomen requiring emergency surgical intervention, studies have disclosed that elevated serum levels of markers such as Lactate, Interleukin-6, C-Reactive Protein and Procalcitonin measured at presentation and peri-operatively may be used as predictors of their evolution [10].

In the early 2010s, Castro et al. stated that LGI can be a marker for the outcome of patients with acute ST-elevation myocardial infarction, and that LGI can be a useful alternative to already established inflammatory markers, hence being a cost-effective and easy-to-perform index [11,12].

The LGI is a combined metric which takes into account white blood cell count and the serum glucose levels whose values are multiplied and their product is then divided by a thousand. The cut-off value is not properly established, but elevated values of over 2000 were positively correlated with negative outcomes and complications in studies conducted to date [13]. Previous studies suggest that it is a potential indicator of SIRS and has proven to be effective in the outcome prediction of patients suffering from stroke and myocardial infarction [12,14]. Moreover, another study conducted by Ramos-Hernández et al. performed on 109 patients with both severe and non-severe forms of COVID-19, concluded that LGI or LGI associated with the respiratory rate was able to predict disease severity and outcome, acting as an independent marker [15].

The potential role of LGI in the prognostication of the severity of diabetic ketoacidosis and microvascular complications was emphasized by a study conducted by Coșarcă et al. which established that LGI can be used as a predictive biomarker aiding in proper risk stratification and tailored treatment and approach [16]. Another study by Mureșan et al. evaluated the utility of LGI in the risk prediction for long-term dysfunction of arteriovenous fistulas in patients with end-stage kidney disease. The research showed promising results, suggesting that LGI may be used as an independent biomarker to improve the management of these patients by early identification of patients at risk and timely intervention [17]. Unlike NLR, MLR, PLR and SII, which are purely leukocyte- or platelet-based ratios reflecting the cellular arm of the inflammatory response, LGI additionally incorporates plasma glucose, capturing the metabolic stress response that accompanies acute surgical illness. Because stress hyperglycemia and leukocytosis are believed to be mechanistically linked through the same neuroendocrine cascade (catecholamines, cortisol, glucagon), LGI may provide a more integrated readout of physiological decompensation than indices derived from the leukocyte differential alone, which could translate into incremental prognostic value in patients with acute surgical abdomen.

To our knowledge, no study has evaluated whether LGI is associated with in-hospital mortality in adult patients with acute surgical abdomen, nor whether it provides prognostic information beyond that already captured by established inflammation-based ratios and routine clinical severity markers (age, SIRS/shock, renal function, comorbidities). The present study was therefore designed to address both questions [18,19,20].

We hypothesized that elevated admission LGI would be independently associated with in-hospital mortality and would show discriminative performance comparable to, or exceeding, that of established inflammation-based ratios. The aim of the current paper is therefore to analyze the utility of LGI in the prediction of in-hospital mortality rates in patients with acute surgical abdomen requiring emergency surgical treatment, and to compare its discriminative and prognostic performance with that of NLR, MLR, PLR and SII.

## 2. Materials and Methods

### 2.1. Study Population

This single-center retrospective cohort study initially included 2334 patients who presented to the Emergency Department (UCPU-SMURD) of the Emergency Clinical County Hospital of Târgu Mureș, Romania, over a two-year timeframe (March 2019 to March 2021), who were diagnosed with acute surgical abdomen and admitted to the Clinical Surgical Department of the same facility. The study period (March 2019–March 2021) encompassed both the pre-pandemic year and the first year of the COVID-19 pandemic; 1261 patients (54.03%) were admitted before and 1073 (45.97%) after March 2020, when national emergency measures were introduced in Romania. Acute surgical abdomen was operationally defined as sudden-onset, severe abdominal pain of less than 72 h’ duration, associated with peritoneal signs and/or radiological/laboratory evidence of an intra-abdominal surgical emergency (e.g., perforated viscus, mesenteric ischemia, acute appendicitis, bowel obstruction, strangulated hernia), confirmed intraoperatively in all cases, as all included patients underwent emergency surgery during the index admission. Of the 2334 patients initially screened, 407 were excluded: 148 for missing data within the charts, 136 for errors in data collection (internally inconsistent, duplicated, or physiologically implausible records, such as laboratory values incompatible with life or recording errors that could not be resolved from the source documents), 55 for age under 18 years, and 68 for transfer to another medical facility, yielding a final cohort of 1927 patients. Baseline age, sex and etiological distribution did not differ significantly between excluded and included patients. Based on the outcome, the study population was divided into two distinct groups: Group 1—patients who were discharged alive after treatment (*n* = 1625, 84.3%) and Group 2—patients who presented in-hospital mortality (*n* = 302, 15.7%)—Figure 1.

As this was a retrospective cohort study, no a priori sample size calculation was performed. Instead, the study included all consecutive adult patients diagnosed with acute surgical abdomen who underwent emergency surgical intervention at our institution during the pre-specified two-year study period (March 2019–March 2021), representing a convenience sample determined by the observation window rather than by a statistical power calculation.

The study was conducted in accordance with the Declaration of Helsinki and was approved by the Ethics Committee of the Emergency Clinical County Hospital of Târgu Mureș (approval no. Ad. 11564/19 May 2023). Given the retrospective design of the study, the requirement for informed consent was waived by the Ethics Committee.

This retrospective cohort study is reported in accordance with the STROBE guidelines (Supplementary Table S1).

### 2.2. Data Collection

The data included in the study were collected from the Emergency Department charts, from the surgical department observation charts and from the hospital information system of the Emergency Clinical County Hospital of Târgu Mureș, Hipocrate^®^ (Romanian Soft Company^©^, Bucharest, Romania), and entered into a Microsoft Excel version 16.100.2 spreadsheet (Microsoft Corporation^®^, Redmond, WA, USA), as numerical and text data. All hematological and biochemical parameters used to calculate the LGI and the inflammation-based indices were obtained from the first blood sample collected at emergency department presentation, before surgery. In routine practice at our department, this sample is drawn at the time of peripheral venous access, usually before or concurrently with the initiation of fluid therapy; however, the exact timing of sampling relative to resuscitation, analgesia or empirical antibiotic therapy was not systematically recorded.

### 2.3. Study Outcome

The primary outcome was all-cause in-hospital mortality; the primary exposure of interest was the admission LGI.

Three hematological ratios (NLR, MLR, PLR) and the systemic immune-inflammation index (SII), calculated as (neutrophil count × platelet count)/lymphocyte count, were derived from the same admission hemogram. The hematological ratios and the SII were computed with all cell counts expressed as ×10^9^/L, whereas LGI was calculated with the leukocyte count expressed as cells/µL, in accordance with the original formula [13]:

$$LGI=\frac{WBC\;\left(\frac{cells}{µl}\right)\times \;plasma\;glucose\;(\frac{mg}{dL})}{1000}$$

The SIRS was defined by the presence of ≥2 of the following: temperature >38 °C or <36 °C, heart rate > 90 bpm, respiratory rate > 20/min or PaCO_2_ < 32 mmHg, and leukocyte count > 12,000/µL, <4000/µL, or >10% immature (band) forms. Shock at admission was defined as systolic blood pressure < 90 mmHg or mean arterial pressure < 65 mmHg with clinical signs of hypoperfusion, and/or the need for vasopressor support.

### 2.4. Statistical Analysis

Statistical analyses were performed using GraphPad Prism version 10 (GraphPad Software, LLC, San Diego, CA, USA). Normality of distribution for all continuous variables was assessed using the Shapiro–Wilk test, complemented by visual inspection of histograms and Q–Q plots; variance homogeneity was evaluated using Levene’s test. Given that none of the continuous variables followed a normal distribution, non-parametric statistical methods were applied throughout the analysis. Continuous variables are therefore presented as median with interquartile range [IQR, 25th–75th percentile]. Between-group differences for continuous variables were evaluated using the Mann–Whitney U test. A two-tailed *p*-value of <0.05 was pre-specified as the threshold for statistical significance. To assess the discriminative capacity of candidate predictors for in-hospital mortality, Receiver operating characteristic (ROC) curve analysis was performed for each of the systemic inflammatory ratio indices. ROC curve analysis was performed for each candidate index using the Wilson/Hybrid Score method for 95% confidence intervals of sensitivity and specificity, as implemented in GraphPad Prism; optimal cut-offs were selected using the Youden index. Multivariable logistic regression was used to identify variables independently associated with in-hospital mortality. Covariate selection combined clinical relevance and univariable significance (*p* < 0.10): age and sex were retained as core demographic covariates; SIRS/shock and renal function markers (urea, creatinine) were included as established indicators of physiological severity; chronic kidney disease and active malignancy were included as comorbidities with an established, independent association with surgical mortality; log-transformed LGI was the primary variable of interest. Leukocyte count and glucose were not entered separately, as both are direct constituents of the LGI. Multicollinearity was assessed by variance inflation factors, model calibration by the Hosmer–Lemeshow test, discrimination by the area under the ROC curve, and explained variance by the Nagelkerke R^2^.

To assess the robustness of the LGI association to etiological heterogeneity, we performed a pre-specified sensitivity analysis. Etiologies were grouped a priori into three clinically defined categories: (1) ischemic/perforative (mesenteric ischemia, perforated viscus), (2) obstructive (bowel obstruction, strangulated hernia), and (3) inflammatory/localized (acute appendicitis, acute cholecystitis, acute diverticulitis, acute pancreatitis, and other/unspecified conditions), the latter serving as the reference category. This grouping was added as a categorical covariate to the primary multivariable logistic regression model, alongside age, sex, SIRS/shock at admission, log-transformed urea and creatinine, chronic kidney disease, active malignancy, and log-transformed LGI.

## 3. Results

A total of 1927 patients admitted with acute surgical abdomen were included in the analysis. Of these, 1625 (84.3%) were successfully discharged alive, while 302 (15.7%) experienced in-hospital death. The main etiologies of acute surgical abdomen were: acute appendicitis (*n* = 551, 28.6%), bowel obstruction (*n* = 459, 23.8%), perforated viscus (*n* = 299, 15.5%), strangulated hernia (*n* = 210, 10.9%), mesenteric ischemia (*n* = 119, 6.2%), acute cholecystitis (*n* = 106, 5.5%), acute pancreatitis (*n* = 77, 4.0%), acute diverticulitis (*n* = 67, 3.5%), and other/unspecified conditions (*n* = 39, 2.0%).

Non-survivors were significantly older than survivors (73 years [IQR25–75: 62–80] vs. 61 years [IQR25–75: 45–72]; *p* < 0.0001).

Baseline characteristics and etiological distribution of survivors and non-survivors are presented in Table 1.

### 3.1. Inflammation-Based Hematological Ratios

All three systemic inflammatory ratio indices were significantly higher in the non-survivors compared to the survivors (all *p* < 0.0001)—Figure 2.

The SII was significantly higher in non-survivors compared with survivors. The median SII value was 2.04 × 10^3^ in Group 2 versus 1.52 × 10^3^ in Group 1, as shown in Figure 3 (*p* < 0.0001).

### 3.2. Hematological Parameters

Several hematological parameters differed significantly between the two groups. Hemoglobin levels were significantly lower in the non-survivors: 11.82 g/dL [IQR25–75: 9.48–13.91] in Group 2 vs. 13.54 g/dL [IQR25–75: 11.87–14.88] in Group 1 (*p* < 0.0001). Hematocrit followed the same pattern: 36.29% [IQR25–75: 29.74–42.13] vs. 40.50% [IQR25–75: 36.2–44.36] (*p* < 0.0001). The red blood cell count was also significantly lower in Group 2: 4.02 × 10^12^/L [IQR25–75: 3.37–4.72] vs. 4.61 × 10^12^/L [IQR25–75: 4.13–5.03] (*p* < 0.0001). The white blood cell count was significantly higher in the non-survivors: 13.77 × 10^3^/µL [IQR25–75: 8.44–20.85] vs. 12.24 × 10^3^/µL [IQR25–75: 8.99–16.24] (*p* = 0.002). Similarly, the absolute neutrophil count was significantly elevated in Group 2: 11.55 × 10^3^/µL [IQR25–75: 6.28–18.11] vs. 9.61 × 10^3^/µL [IQR25–75: 6.34–13.41] (*p* = 0.0001). The absolute lymphocyte count was significantly lower in the non-survivors: 1.14 × 10^3^/µL [IQR25–75: 0.69–1.87] vs. 1.45 × 10^3^/µL [IQR25–75: 0.99–2.06] (*p* < 0.0001). In contrast, the monocyte count and platelet count did not differ significantly between the two groups (both *p* > 0.05).

### 3.3. Biochemical Parameters

Significant differences between groups were observed for several biochemical markers. Plasma glucose at admission was higher in the non-survivors: 130 mg/dL vs. 117 mg/dL (*p* = 0.0004). Urea was markedly elevated in Group 2: 70.6 mg/dL vs. 33.4 mg/dL (*p* < 0.0001). Serum creatinine was likewise significantly higher in patients who died in hospital: 1.43 mg/dL vs. 0.85 mg/dL (*p* < 0.0001).

Aspartate aminotransferase (AST) was significantly elevated in Group 2: 31 U/L vs. 22 U/L (*p* < 0.0001). Alanine aminotransferase (ALT) did not reach statistical significance: median 24 U/L vs. 23 U/L (*p* = 0.43).

A summary of the biochemical between-group comparisons is presented in Table 2.

### 3.4. ROC Curve Analysis of Inflammatory Ratio Indices

ROC curve analysis was performed to evaluate the discriminative capacity of NLR, MLR and PLR for in-hospital mortality (Figure 4). Discrimination was poor for all three ratios, with AUCs between 0.580 and 0.597 and lower 95% confidence limits between 0.541 and 0.558, only marginally above the no-discrimination value of 0.5.

PLR showed poor discriminative ability, with an AUC of 0.593 (95% CI 0.554–0.632) and an optimal cut-off value of > 206.7. At this threshold, PLR achieved a sensitivity of 59.93% (95% CI: 54.32–65.30%) and a specificity of 59.38% (95% CI: 56.98–61.75%), corresponding to an LR+ of 1.48 and an LR− of 0.68. NLR showed a similar performance, with an AUC of 0.597 (95% CI 0.558–0.636) and an optimal cut-off of >7.545, yielding a sensitivity of 58.28% (95% CI: 52.65–63.70%) and a specificity of 57.05% (95% CI: 54.63–59.43%), and an LR+ of 1.36 and an LR− of 0.73. MLR demonstrated the lowest discriminative power (AUC = 0.580; 95% CI 0.541–0.618) at a cut-off of > 0.585, it provided a sensitivity of 57.28% (95% CI: 51.65–62.74%) and a specificity of 58.77% (95% CI: 56.36–61.14%), with an LR+ of 1.39 and an LR− of 0.727.

The SII demonstrated an AUC of 0.581 (95% CI: 0.542–0.620), indicating limited discriminative ability. At the optimal cut-off value of >3.29 × 10^3^, SII achieved a sensitivity of 36.4% (95% CI 31.18–41.97%) and a specificity of 80.7% (95% CI 78.71–82.55%), corresponding to an LR+ of 1.89 and an LR− of approximately 0.79—Figure 5.

A comparative summary of the discriminative performance of NLR, MLR, PLR, and SII for predicting in-hospital mortality, including AUC values with 95% confidence intervals and the corresponding optimal cut-offs, sensitivity, specificity, and likelihood ratios, is presented in Table 3.

### 3.5. The Leuko-Glycemic Index

The LGI values differed significantly between the two groups, with significantly higher values in Group 2 significant higher values (1986 [IQR25–75: 1263–3113] vs. 1568 [IQR25–75: 980–2083]; *p* < 0.0001)—Figure 6.

The LGI demonstrated an area under the curve (AUC) of 0.646 (95% CI: 0.607–0.685), indicating modest discriminative ability, albeit the highest among the indices tested. At the optimal cut-off value of >1665, LGI achieved a sensitivity of 60.17% (95% CI: 55.02–65.10%) and a specificity of 57.73% (95% CI: 55.35–60.07%), corresponding to an LR+ of 1.42 and an LR− of 0.69—Figure 7.

Pairwise comparison of ROC curves using the DeLong test showed that LGI had a significantly higher AUC than MLR (Δ AUC = 0.084, z = 3.75, *p* = 0.0002), PLR (Δ AUC = 0.091, z = 3.71, *p* = 0.0002), and SII (Δ AUC = 0.096, z = 4.14, *p* < 0.0001), and a nominally significant difference versus NLR (Δ AUC = 0.048, z = 2.03, *p* = 0.042). No statistically significant differences were observed among NLR, MLR, PLR, and SII (all *p* > 0.05), except for the NLR vs. SII comparison, which reached borderline significance (*p* = 0.034). The difference between LGI and NLR was small in absolute terms (ΔAUC 0.048) and would not remain significant after correction for the ten pairwise comparisons performed (Bonferroni-adjusted threshold *p* < 0.005); it should therefore not be interpreted as evidence of a clinically meaningful superiority of LGI over NLR.

To assess the stability of the LGI cut-off and correct for potential overfitting inherent to threshold derivation within the same cohort, internal validation was performed using bootstrap resampling (2000 resamples with replacement). The bootstrap-corrected AUC was 0.649 (95% CI 0.615–0.680), closely matching the apparent AUC (optimism = 0.0004), indicating minimal overfitting of overall discrimination. The optimal cut-off of 1665 was confirmed as the median value across bootstrap resamples; however, its 95% CI (1140–2455) was considerably wide, reflecting instability of the point estimate, particularly given the pronounced imbalance between survivors and non-survivors. Given this instability, the proposed cut-off should be regarded as an exploratory, hypothesis-generating threshold rather than an established clinical decision point, warranting external validation in independent, prospective cohorts before any consideration for clinical implementation.

### 3.6. Multivariable Logistic Regression

In multivariable logistic regression, log-transformed LGI remained independently associated with death after adjustment, with a 44% increase in the odds of in-hospital mortality per 1 SD increment (OR 1.44, 95% CI 1.20–1.73, *p* = 0.0001). The variable most strongly associated with in-hospital mortality was SIRS or shock at admission (OR 7.05, 95% CI 4.71–10.57, *p* < 0.0001), followed by markers of renal dysfunction, log urea (OR 5.27, 95% CI 4.07–6.84 per 1 SD) and log creatinine (OR 4.62, 95% CI 3.52–6.06 per 1 SD), both *p* < 0.0001. Chronic kidney disease (OR 3.52, 95% CI 2.28–5.42) and malignancy (OR 3.42, 95% CI 1.97–5.92) each more than tripled the adjusted odds of death (both *p* < 0.0001), while each additional decade of age conferred a 51% increase (OR 1.51, 95% CI 1.34–1.69, *p* < 0.0001). Male sex showed a weaker association that reached nominal significance, with a lower confidence limit close to unity (OR 1.47, 95% CI 1.04–2.09, *p* = 0.031). Leukocyte count and glucose were not entered as separate covariates, as both are constituents of the LGI product and their reintroduction alongside the index produced severe collinearity (variance inflation factors 13.9 and 12.1, respectively, with 30.2 for log LGI); in the final model all variance inflation factors were below 1.7. The model showed moderate-to-good discrimination (AUC 0.765, 95% CI 0.74–0.79), adequate calibration (Hosmer–Lemeshow *p* = 0.70) and a Nagelkerke R^2^ of 0.25, with 38 events per variable. Known diabetes mellitus was present in 160 (8.3%) patients of the cohort (133, 8.2% in Group 1 vs. 27, 8.9% in Group 2; *p* = 0.65). A sensitivity analysis adjusting the multivariable model for diabetes status did not materially change the association between log-transformed LGI and in-hospital mortality (adjusted OR 1.43, 95% CI 1.18–1.72, *p* = 0.0001); excluding patients with known diabetes yielded similar results (AUC 0.649) (Figure 8).

Table 4 summarizes the full multivariable model, in which log-transformed LGI remained independently associated with in-hospital mortality after adjustment for age, sex, SIRS/shock at admission, renal function, chronic kidney disease, and active malignancy.

In the etiology-adjusted sensitivity model (10 predictor terms, 291 events, approximately 29 events per variable), log-transformed LGI remained independently associated with in-hospital mortality (adjusted OR 1.23, 95% CI 1.08–1.41, *p* = 0.002). Compared with the inflammatory/localized reference group, the ischemic/perforative group showed significantly higher adjusted odds of death (OR 1.81, 95% CI 1.30–2.54, *p* = 0.001), while the obstructive group did not differ significantly from the reference (OR 1.09, 95% CI 0.79–1.50, *p* = 0.59). All other covariates retained directionally consistent associations with the primary model (SIRS/shock OR 3.04; log urea OR 1.75; log creatinine OR 1.78; chronic kidney disease OR 1.59; active malignancy OR 2.08; age per decade OR 1.21; all *p* < 0.05). Model discrimination (AUC 0.77) and calibration (Hosmer–Lemeshow *p* = 0.99) were adequate, and all variance inflation factors remained below 1.7 (maximum 1.2), indicating no meaningful multicollinearity introduced by the etiology grouping.

## 4. Discussion

The LGI, a relatively novel inflammatory biomarker, has been identified as a key marker in other conditions, such as stroke, myocardial ischemia, acute pulmonary embolism, peripheral artery disease, gestational diabetes, and atherosclerosis [21,22,23,24,25]. To our knowledge, there are currently no published studies that have investigated the role of LGI in the prediction of mortality in patients with acute surgical abdomen. Acute surgical abdomen is a surgical emergency carrying high morbidity and mortality rates, requiring immediate surgical intervention [6,8]. The current study’s aim was to evaluate the association between admission LGI and in-hospital mortality rates of patients with acute surgical abdomen and the values of LGI and other inflammatory markers, by analyzing the data collected from a total of 1927 patients, over the course of two years. The observed in-hospital mortality of 15.7% exceeds the 2–14% range generally reported for acute surgical abdomen [8], most likely reflecting the case mix of a single tertiary emergency referral center, the composite inclusion of high-severity etiologies (e.g., mesenteric ischemia, perforation, septic shock) alongside lower-severity presentations, and the requirement for emergency surgical intervention in all included patients. An additional contributing factor may be the overlap of the study period with the first year of the COVID-19 pandemic. Pandemic-related restructuring of hospital circuits, reduced elective activity, and patient reluctance to seek emergency care have been associated with delayed presentation and a higher proportion of advanced, complicated forms of acute surgical abdomen, including perforation and bowel ischemia [6,7]. Such a shift in case mix would be expected to raise both baseline inflammatory–metabolic activation and observed mortality.

In accordance with other studies published on this topic, age was independently associated with in-hospital death within the cohort. A systematic review conducted by Cooper Z. et al. concluded that the 30-day mortality risk in patients undergoing emergency abdominal surgery ranged between 25% and 50%, with odds increasing in patients over the age of 65 [26]. Moreover, the chances for elderly patients to develop postoperative complications like pneumonia, urinary tract infections, delirium, acute kidney injury, and progressive loss of physical and cognitive abilities are much higher than in younger patients [27].

In our study, routine hematological parameters measured at presentation were observed to be strongly associated with in-hospital mortality. The parameters that proved to be significant within the non-survivors were low levels of hemoglobin, hematocrit, red blood cells, and lymphocytes, and high absolute numbers of leukocytes and neutrophils, while monocyte and platelet counts did not differ significantly between groups. This is consistent with previous reports that peri-operative anemia is associated with a longer hospital stay, more infectious complications, and higher short- and long-term mortality. Whether pre-operative correction of anemia would improve outcomes cannot be inferred from our data; this hypothesis would need to be tested in prospective interventional studies, particularly given that anemia may equally act as a marker of underlying disease severity rather than as a modifiable causal factor [28,29].

Regarding the inflammatory response, the study we conducted identified that absolute leukocyte and neutrophil counts were increased significantly in non-survivors, which emphasizes the role of both as independent markers of systemic inflammation. Moreover, the perioperative elevated neutrophil-to-lymphocyte ratio was significantly associated with negative outcomes within the studied group, mainly due to increased values of the absolute number of neutrophils and decreased absolute numbers of lymphocytes. This is consistent with previous research that identified the NLR as a good predictor of peri-operative complications, in-hospital death, and infections of the surgical wounds [30].

Several biochemical markers were also found to correlate with negative outcomes in the patients included in our study. Among these, admission plasma glucose was higher in non-survivors, which is in accordance with the notion of stress hyperglycemia, which can occur in the context of acute surgical pathologies. Previous research has found a strong link between peri-operative hyperglycemia, post-operative complications (mostly infectious) prolonged hospital stay, and higher mortality rates, in both patients previously diagnosed with diabetes and normoglycemic patients [31]. Higher glucose levels at admission demonstrate the consequence of counter-regulatory hormones, systemic inflammation and insulin resistance, indicating greater physiological stress in the context of acute surgical abdomen, potentially contributing to risk assessment in these patients [32].

Renal impairment markers showed important and significant differences between our two study groups, with both urea and creatinine being higher in the non-survivors. A large retrospective cohort study conducted by Mikkelsen et al. concluded that peri-operative acute kidney injury significantly increased the 90-day mortality rates in patients undergoing major abdominal surgery, which is consistent with the results of our study [33]. Moreover, another study found serum urea values to influence 30-day mortality rates in patients undergoing major abdominal surgery, but serum creatinine levels did not prove to be associated with it [34]. Nonetheless, another study suggests that the presence of acute kidney injury (AKI) at admission in patients admitted for infectious pathologies does not predict long-term mortality, since no prediction as to whether the AKI will only be transient or non-recoverable can be made [35].

The finding that LGI was significantly elevated in patients who died in the hospital is consistent with its previously reported utility across a broad spectrum of acute cardiovascular and non-cardiovascular conditions. Beyond its established role in acute myocardial infarction [11,12] and stroke [14], LGI has more recently been evaluated as a prognostic tool in postoperative cardiac surgery patients. Seoane et al. demonstrated that high LGI values were independently associated with in-hospital mortality, low cardiac output syndrome, and acute kidney injury in patients undergoing coronary artery bypass grafting (CABG), establishing a cut-off value of 2000 as the best predictor of the primary composite endpoint [13]. These data are consonant with the cut-off of 1665 identified in our cohort, suggesting that, despite the different clinical settings, the pathophysiological substrate linking immune-metabolic dysregulation to adverse outcomes may share common mechanisms regardless of the triggering condition.

The biological rationale underlying the prognostic value of LGI resides in the bidirectional interaction between leukocytosis and hyperglycemia in the context of systemic inflammation. Acute surgical pathologies trigger a well-characterized neuroendocrine and immune response, leading to the release of counter-regulatory hormones (including cortisol, glucagon, and catecholamines), which drive hepatic glucose production, peripheral insulin resistance, and immune cell mobilization simultaneously [36]. This stress hyperglycemia, occurring independently of pre-existing diabetes, has been repeatedly associated with impaired neutrophil and monocyte bactericidal function, increased susceptibility to surgical site infections, and prolonged organ dysfunction [36]. The concurrent leukocytosis reflects a systemic inflammatory response that, when sustained or disproportionate, may itself contribute to tissue injury through oxidative stress, endothelial dysfunction, and dysregulated cytokine release [37]. LGI elegantly captures both of these axes in a single, easily calculable parameter, which may partly explain the association observed in the current cohort.

The numerically highest AUC of LGI (0.646) compared with NLR (0.597), MLR (0.580), and PLR (0.593) in our cohort merits attention. While all four indices reflect facets of systemic inflammation, LGI integrates a metabolic parameter, blood glucose, which adds independent pathophysiological information beyond the leukocyte differential alone. The utility of NLR and PLR as prognostic tools in emergency abdominal surgery has been previously documented [32], and a retrospective study by El-Menyar et al. found that PLR, but not NLR alone, independently predicted mortality in abdominal trauma patients [38]. In the context of non-traumatic acute surgical abdomen, our results suggest that the composite metabolic-inflammatory index represented by LGI may capture additional prognostic information not fully reflected by hematological ratios alone, particularly in a mixed etiological cohort encompassing perforation, ischemia, obstruction, and infectious pathologies. It is important to distinguish association from discrimination and clinical utility: the independent association of log-transformed LGI with mortality indicates that it carries information not explained by the other covariates, but its modest standalone AUC does not support individual risk prediction, and any effect on clinical decision-making would require prospective evaluation. The sensitivity analysis indicates that the independent association between LGI and mortality is not simply an artifact of etiological case-mix: after accounting for the broad clinical category of the underlying surgical pathology, the LGI effect size remained essentially unchanged from the primary model, while the etiology grouping itself confirmed that ischemic/perforative presentations carry substantially higher adjusted mortality risk than inflammatory/localized ones.

These data do not support the use of LGI as a triage instrument, and in particular, not to limit, withhold or withdraw active treatment. First, a group-level association does not translate into reliable individual prediction: with likelihood ratios close to unity, a value above the proposed threshold raised the estimated probability of death only from 15.7% to approximately 21%, and a value below it lowered it only to approximately 11%. Second, the threshold was unstable on internal validation (95% CI 1140–2455). Third, the leukocytosis and hyperglycemia captured by the index largely reflect a potentially reversible stress response that timely source control and resuscitation are intended to correct; a high LGI identifies a more physiologically deranged patient, not one in whom intervention is futile. Whether LGI could serve as an automatically generated flag prompting earlier senior review or closer monitoring is a hypothesis that requires prospective validation; it is not a recommendation arising from the present study.

The SII, which incorporates platelet count as a third cellular component, showed discrimination comparable to the hematological ratios (AUC 0.581) and below that of LGI. Notably, however, SII operated at a different point on the sensitivity–specificity trade-off: at its optimal threshold, it was considerably more specific (80.7%) but far less sensitive (36.4%) than LGI, yielding the highest positive likelihood ratio of all indices tested (1.89 vs. 1.42 for LGI). Even so, a value above the SII threshold would raise the estimated probability of death only from 15.7% to approximately 26%, so neither index is suitable as a stand-alone rule-in or rule-out test. The different operating points suggest only that the two indices may convey partly complementary information, a hypothesis that warrants formal evaluation in larger series.

LGI has also demonstrated prognostic utility in a range of non-surgical acute conditions, including COVID-19, where Ramos-Hernández et al. showed that LGI combined with respiratory rate yielded an AUC of 0.894 for predicting disease severity, outperforming either parameter used in isolation [15]. More recently, Wang et al. confirmed the association of LGI with in-hospital mortality in critically ill stroke patients using a large multicenter database, reporting significant differences between survivors and non-survivors across both ischemic and hemorrhagic subtypes [21]. Similarly, a study by Nafea et al. evaluated LGI in acute methanol poisoning and demonstrated an AUC of 0.808 for predicting in-hospital mortality, identifying the index as a cost-effective and accessible triage tool in the emergency setting [36]. Collectively, these findings reinforce the concept that LGI operates as a trans-diagnostic marker of physiological decompensation, which lends further biological plausibility to its utility in acute surgical abdomen.

Perioperative hyperglycemia, regardless of pre-existing diabetic status, is independently associated with surgical site infections, prolonged ICU stay, and higher mortality in both cardiac and general surgical populations [31,32,37], consistent with the significant admission glucose difference observed between survivors and non-survivors in our cohort and supporting blood glucose as a physiologically meaningful component of the LGI formula. This aligns with emerging evidence that combined immunometabolic indices may add incremental prognostic value over classical severity scores: elevated LGI has been linked to diabetic ketoacidosis severity and microvascular complications [16] and to symptomatic atherosclerotic disease in young patients [25], reinforcing its translational relevance across the acute-to-chronic disease spectrum.

From a clinical and translational perspective, a particularly relevant contribution to the LGI literature comes from Banceu et al., who evaluated mortality outcomes in aortic dissection [39]. This study highlights the importance of resource availability and timing of presentation in determining surgical outcomes in cardiovascular emergencies, underscoring that biomarker research must be contextualized within the broader clinical environment.

It should be noted that the optimal cut-off value for LGI in predicting mortality in acute surgical abdomen has not been previously defined, and our study provides the first such threshold (1665). The cut-off values identified in other acute conditions vary considerably: Castro et al. proposed a value above 2000 in acute STEMI patients [11], while Seoane et al. applied the same threshold in post-CABG patients [13], Qi et al. reported cut-offs ranging from 1448 to 1760 depending on diabetic status in acute myocardial infarction patients [12], and You et al. used a cut-off of 1755 in acute ischemic stroke [14]. The lower threshold observed in our cohort may reflect the heterogeneity of the acute surgical abdomen population, in which milder degrees of immune-metabolic activation may already carry prognostic significance compared with purely ischemic or cardiogenic conditions. Additionally, the absence of large, previously published cohorts for this indication precludes external validation of our threshold, and the identified cut-off should be interpreted with the appropriate degree of caution pending replication in independent series.

An important consideration in interpreting the present findings is the heterogeneity of the acute surgical abdomen as a clinical entity. The included patients presented with a wide array of underlying pathologies, ranging from perforated viscera and acute appendicitis to mesenteric ischemia and strangulated hernias, each with distinct inflammatory and metabolic profiles. This inherent heterogeneity is simultaneously a strength, in that the LGI demonstrated predictive utility across this diverse population, and a limitation, as subgroup analyses by etiology were not performed. Previous studies have highlighted that the leukocyte differential may differ meaningfully between perforative and obstructive etiologies [18], while the glycemic response may be more pronounced in ischemic conditions due to the greater degree of counterregulatory hormone activation [36]. Future analyses stratified by underlying diagnosis may therefore refine the clinical applicability of LGI thresholds in this patient population.

While the primary objective of this study was to evaluate LGI as a mortality predictor, the observed differences in multiple inflammatory and biochemical parameters underscore that in-hospital death in acute surgical abdomen is a multifactorial event, likely driven by the interaction between the severity of the acute insult, the adequacy of the host inflammatory response, and pre-existing organ reserve. Age, which was independently associated with mortality in our cohort, has been consistently identified in the surgical literature as a dominant risk factor for adverse outcomes following emergency abdominal surgery, particularly in patients over 65, among whom the incidence of post-operative complications including pneumonia, delirium, acute kidney injury, and prolonged functional decline is substantially elevated [26,27]. The integration of LGI with established clinical scores and demographic variables in future multivariable prediction models may therefore yield tools with superior calibration and discrimination compared with any individual biomarker.

The present study should be interpreted in light of several limitations. Its retrospective, single-center design limits the extrapolation of the findings to larger populations, and the results require external validation in prospective multicenter cohorts with standardized data collection protocols. The absence of serial LGI measurements precluded assessment of the dynamic changes in the index over the clinical course, which may carry additional prognostic information beyond the admission value alone. Although the multivariable model adjusted for age, sex, SIRS/shock, renal dysfunction, chronic kidney disease and malignancy, it could not account for corticosteroid use, prior antibiotic therapy or nutritional status, all of which may influence both leukocyte count and glycemia independently of the acute surgical condition. The composite etiological nature of the acute surgical abdomen cohort, while reflective of real-world emergency practice, introduces clinical heterogeneity that limits inferences about the optimal LGI threshold in specific diagnostic subgroups. Standardized severity scores (e.g., APACHE II, SOFA) or established surgical risk scores were not systematically recorded, precluding assessment of the incremental value of LGI over currently used clinical tools. The LGI cut-off of 1665 was derived and evaluated in the same cohort, creating a risk of optimism in the reported sensitivity and specificity; this threshold should be regarded as exploratory and hypothesis-generating rather than a validated clinical cut-off, and future work should test prespecified thresholds from prior literature ideally, external validation in an independent cohort. The pronounced imbalance between survivors (*n* = 1625) and non-survivors (*n* = 302), while not directly biasing AUC, may affect the precision and stability of the derived cut-off and should be considered when interpreting the diagnostic metrics reported in the present work. In addition, the exclusion of 407 patients, predominantly because of missing or erroneous chart data, may have introduced selection bias, although included and excluded patients did not differ in age, sex or etiological distribution. Internal validation was limited to bootstrap resampling within the derivation cohort; no external validation was performed. Interventions performed prior to blood sampling (e.g., fluid resuscitation, empirical antibiotic therapy, analgesia) were not systematically recorded and could have influenced admission leukocyte and glucose values in a subset of patients, potentially attenuating or exaggerating the observed associations.

The study period spanned the transition into the COVID-19 pandemic, and pandemic-related changes in referral patterns, time to presentation, and case severity may have influenced both the observed mortality rate and the distribution of the inflammatory and metabolic parameters analyzed; SARS-CoV-2 status was not systematically recorded, and the derived LGI threshold should therefore be re-examined in cohorts recruited outside this period.

Future prospective studies with etiological stratification, integration of LGI into clinical scoring systems, and evaluation of serial measurements are needed to fully establish the role of this index in the triage and management of patients with acute surgical abdomen, and to determine whether LGI-guided interventions, such as early glucose control protocols or targeted anti-inflammatory strategies, can translate the biomarker’s prognostic utility into tangible clinical benefit. These remain hypotheses for future prospective research; the present study did not evaluate any LGI-guided intervention, and no claim regarding personalized emergency management, glycemic control, or anti-inflammatory therapy can be made on the basis of the current, purely observational, associative data.

## 5. Conclusions

In this single-center retrospective cohort of patients undergoing emergency surgery for acute surgical abdomen, a higher admission LGI was associated with in-hospital mortality independently of the clinical covariates examined and discriminated mortality at least as well as the inflammation-based ratios tested. However, its standalone discrimination was modest, and its optimal threshold unstable, so LGI is not suitable for triage or for individual treatment decisions. At present, LGI should be regarded as an exploratory, low-cost marker of immune-metabolic stress, and prospective multicenter studies with etiological stratification, comparison against established severity scores, and external validation are required before any clinical application can be considered.

## Figures and Tables

**Figure 1:**
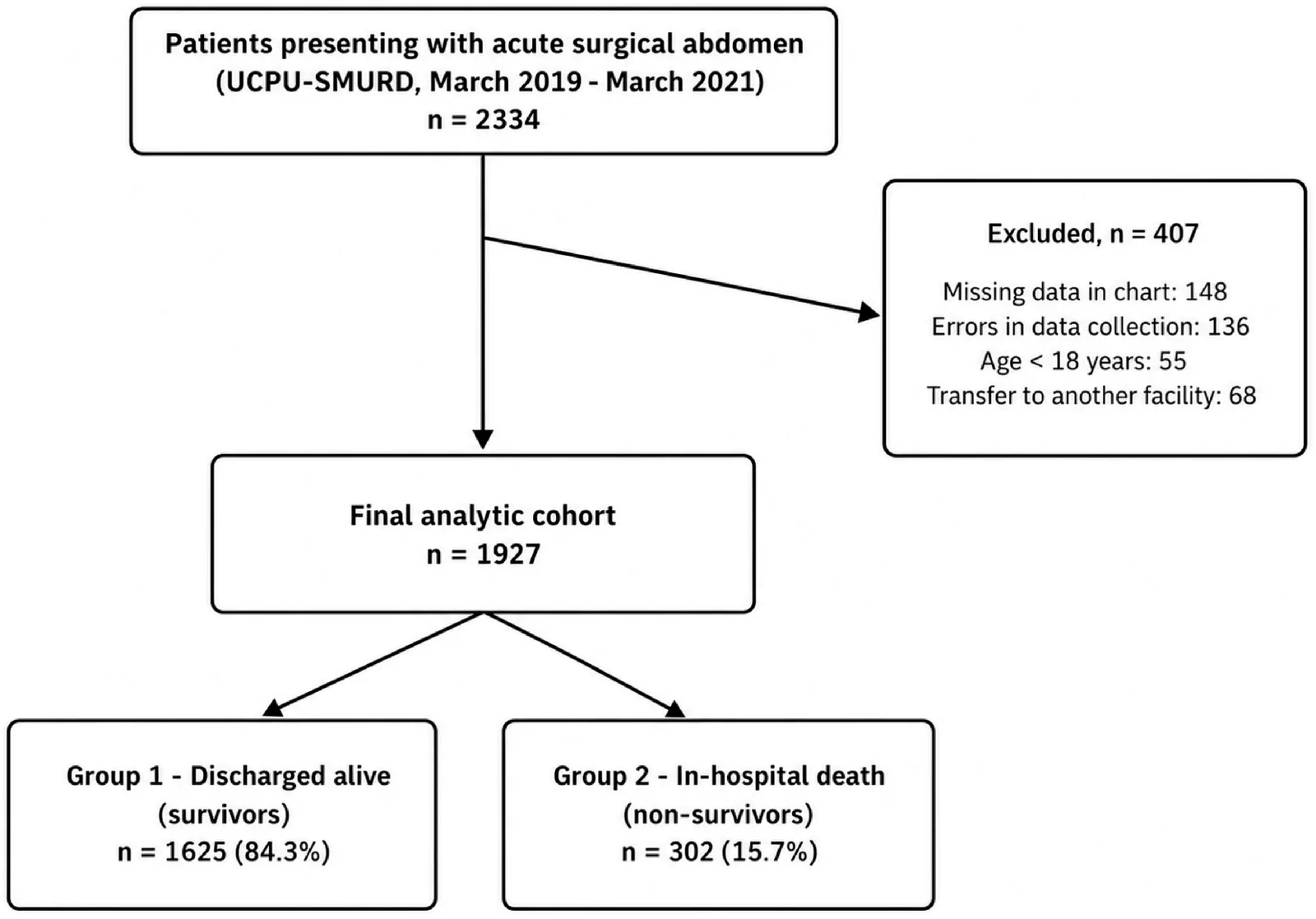
Patient flow diagram: study screening, exclusions, and final analytic cohort stratified by in-hospital survival status.

**Figure 2:**
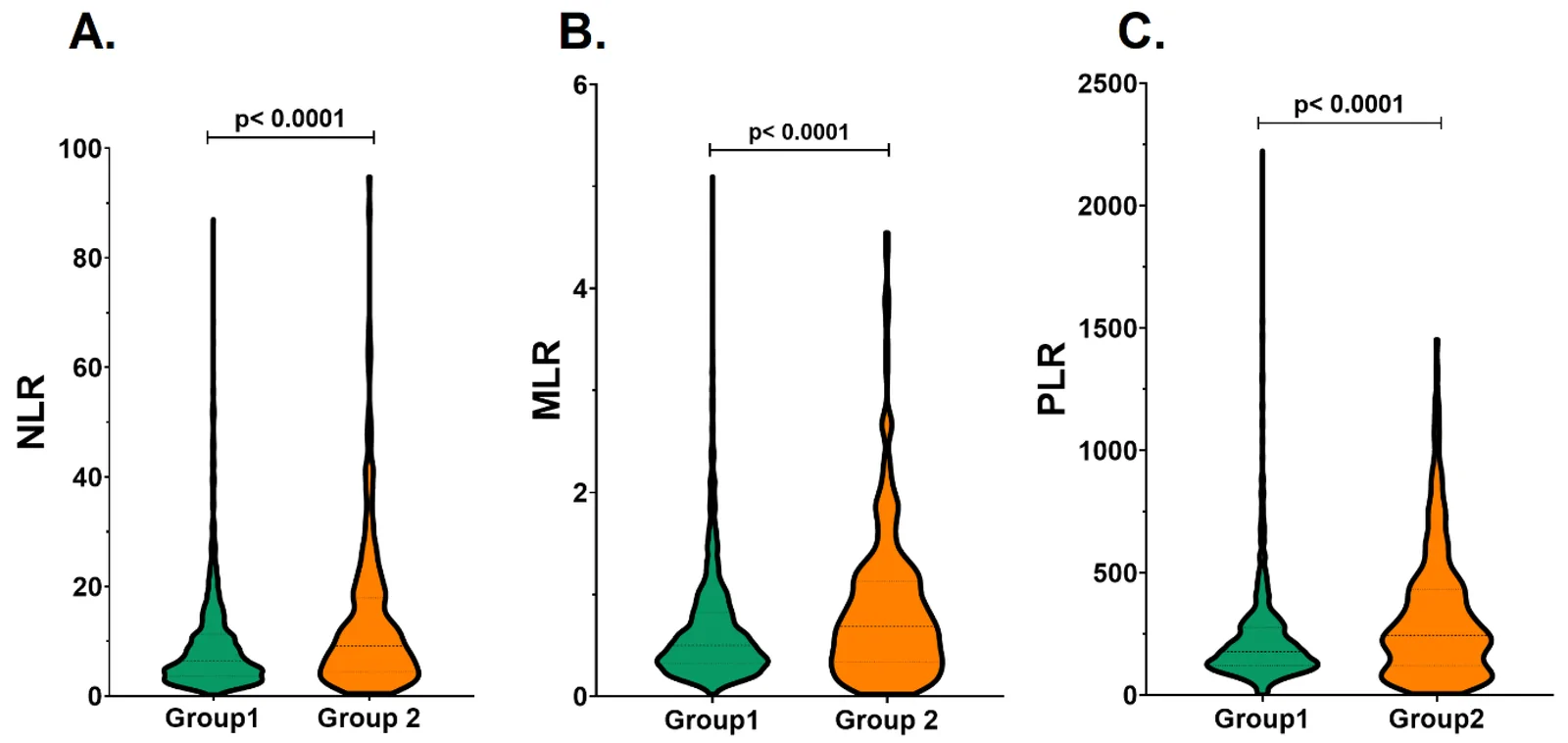
Comparison of inflammation-based hematological ratios between study groups. Violin plots illustrate the distributions of the (**A**) neutrophil-to-lymphocyte ratio, (**B**) monocyte-to-lymphocyte ratio, and (**C**) platelet-to-lymphocyte ratio in the two groups. Data are presented as violin plots; the dashed line represents the median and the dotted lines represent the interquartile range (25th and 75th percentiles).

**Figure 3:**
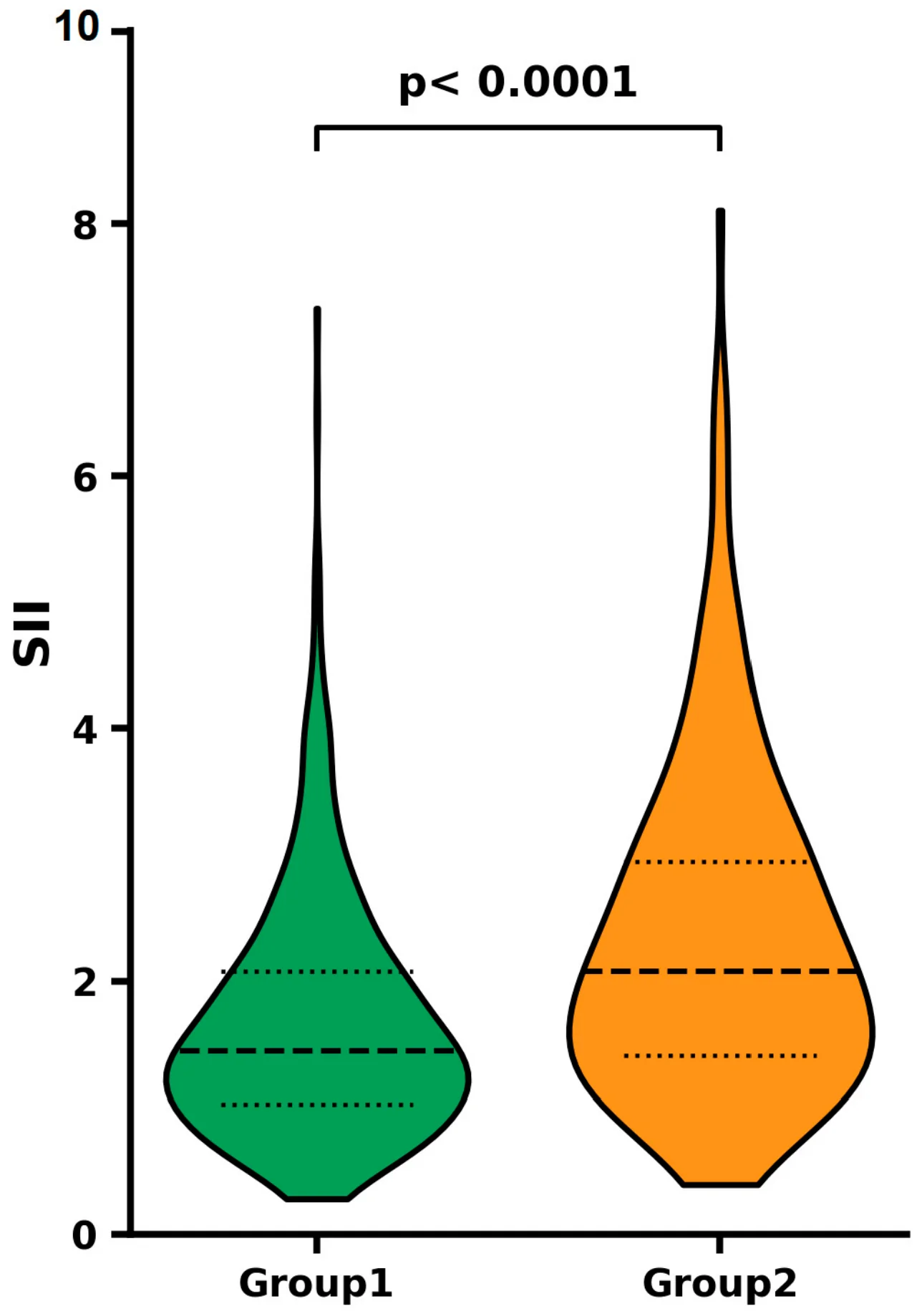
Comparison of SII between study groups. Data are presented as violin plots; the dashed line represents the median and the dotted lines represent the interquartile range (25th and 75th percentiles).

**Figure 4:**
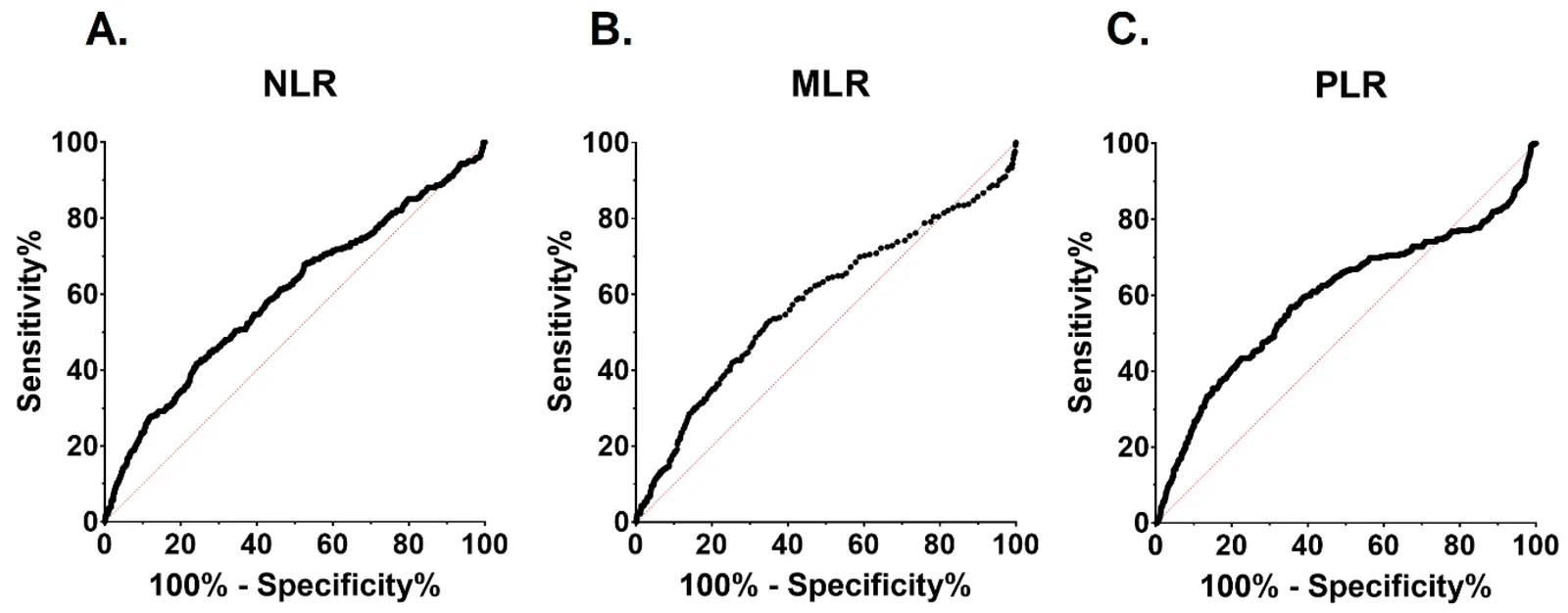
Receiver operating characteristic (ROC) curves for inflammation-based hematological indices. Panel (**A**) shows the ROC curve for the neutrophil-to-lymphocyte ratio, (**B**) for the monocyte-to-lymphocyte ratio, and (**C**) for the platelet-to-lymphocyte ratio.

**Figure 5:**
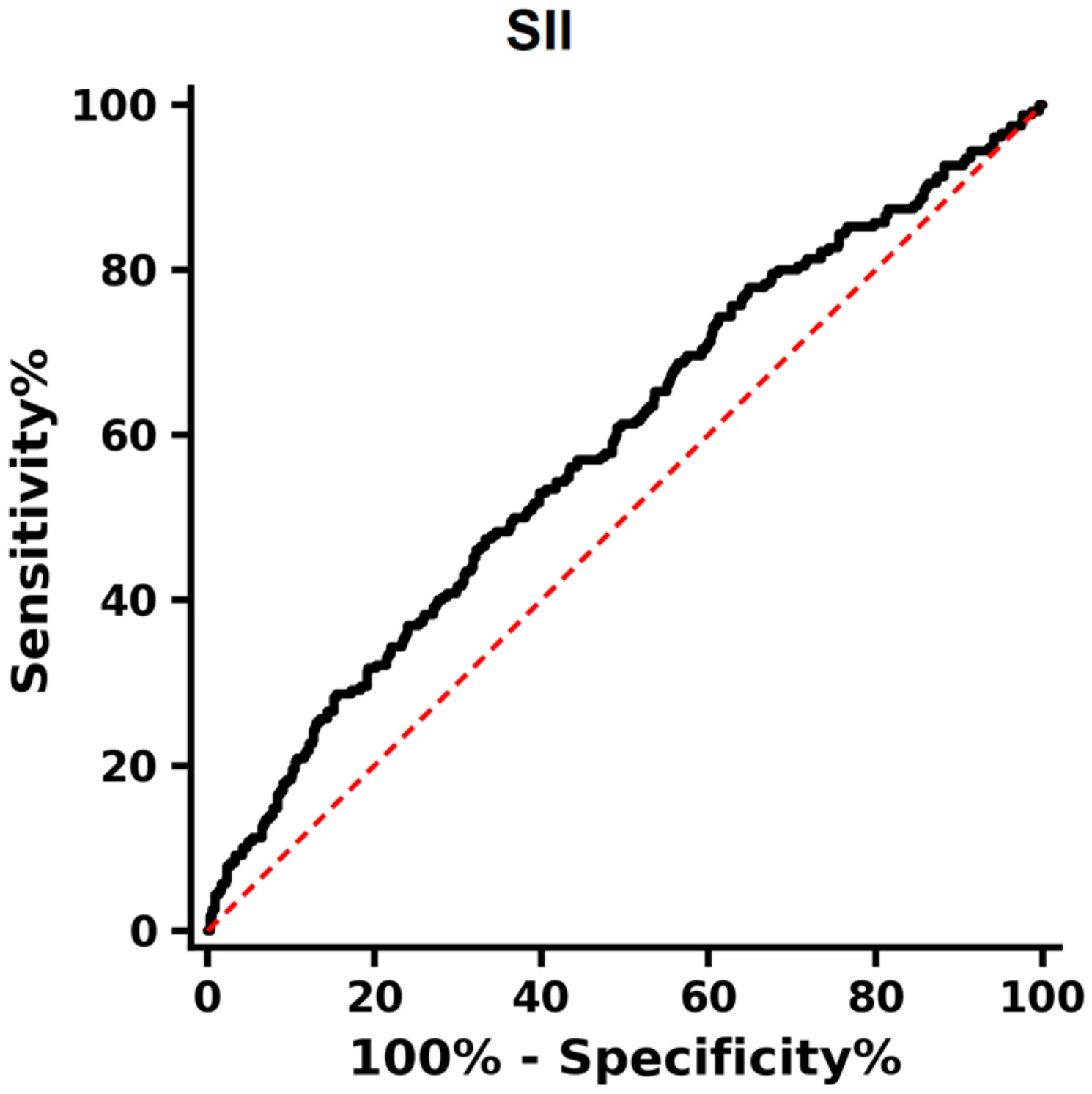
Receiver operating characteristic (ROC) curve of the SII for prediction of the study outcome.

**Figure 6:**
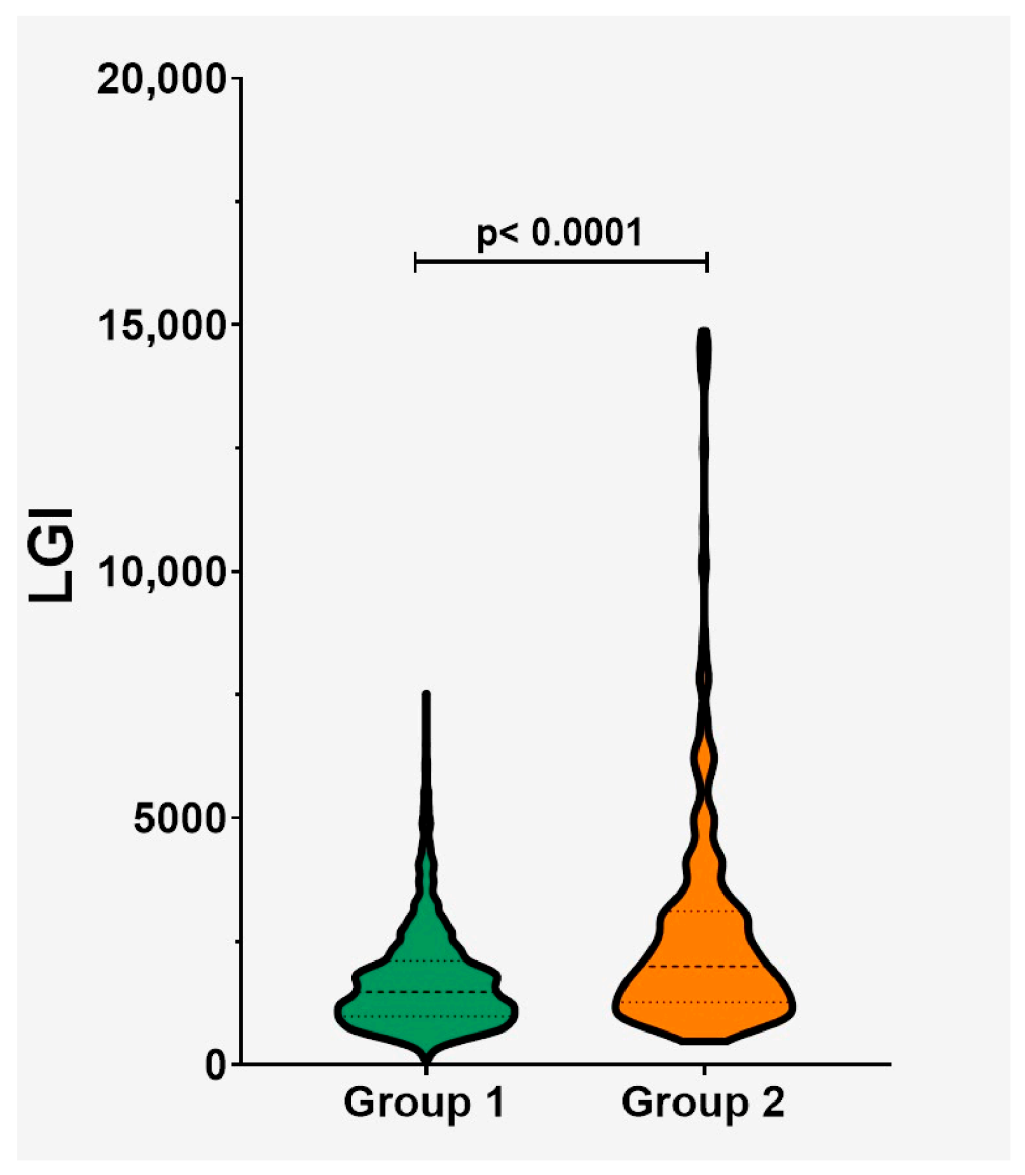
Distribution of the LGI in the study groups. Data are presented as violin plots; the dashed line represents the median and the dotted lines represent the interquartile range (25th and 75th percentiles).

**Figure 7:**
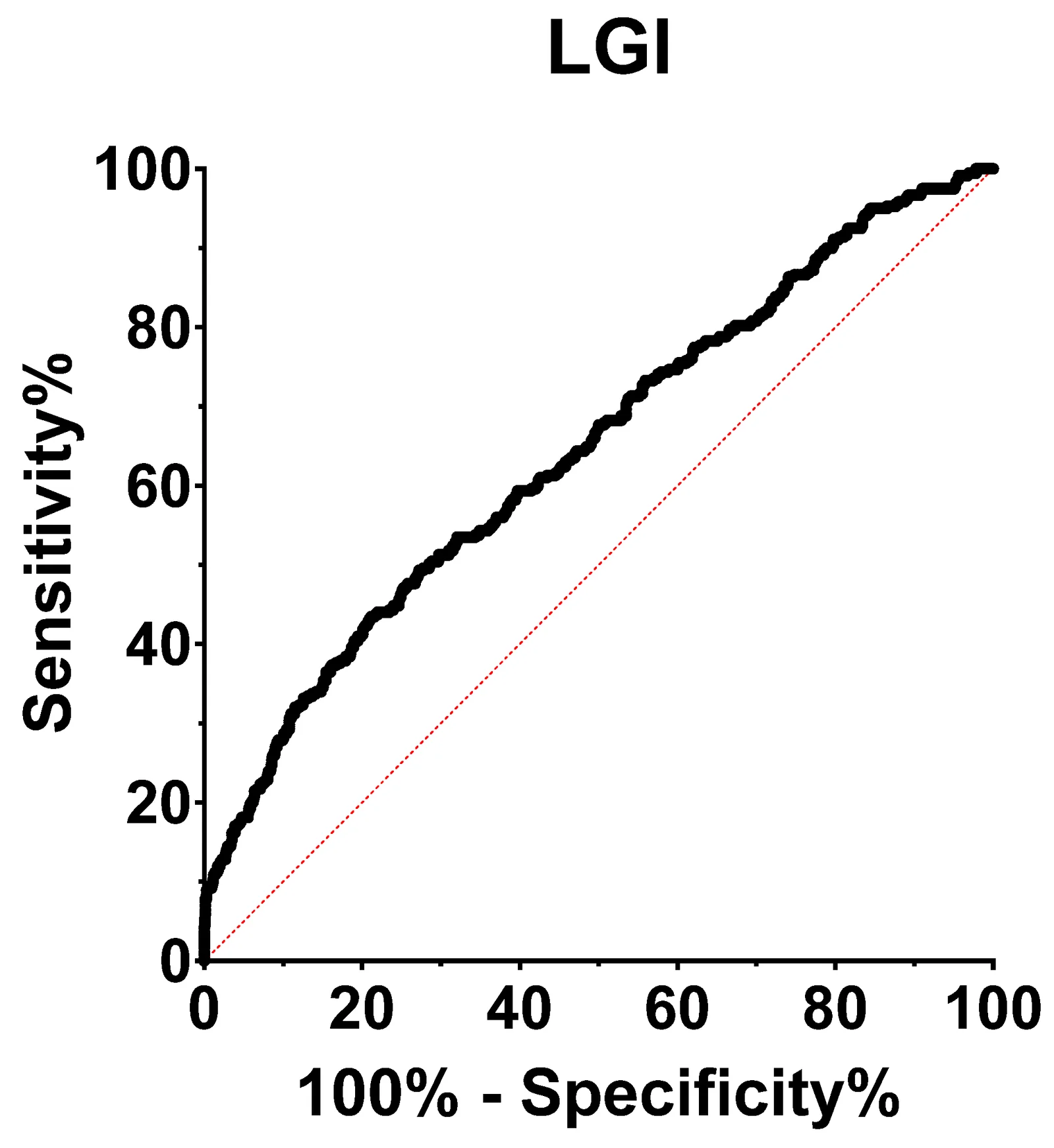
Receiver operating characteristic (ROC) curve of the LGI for prediction of the study outcome.

**Figure 8:**
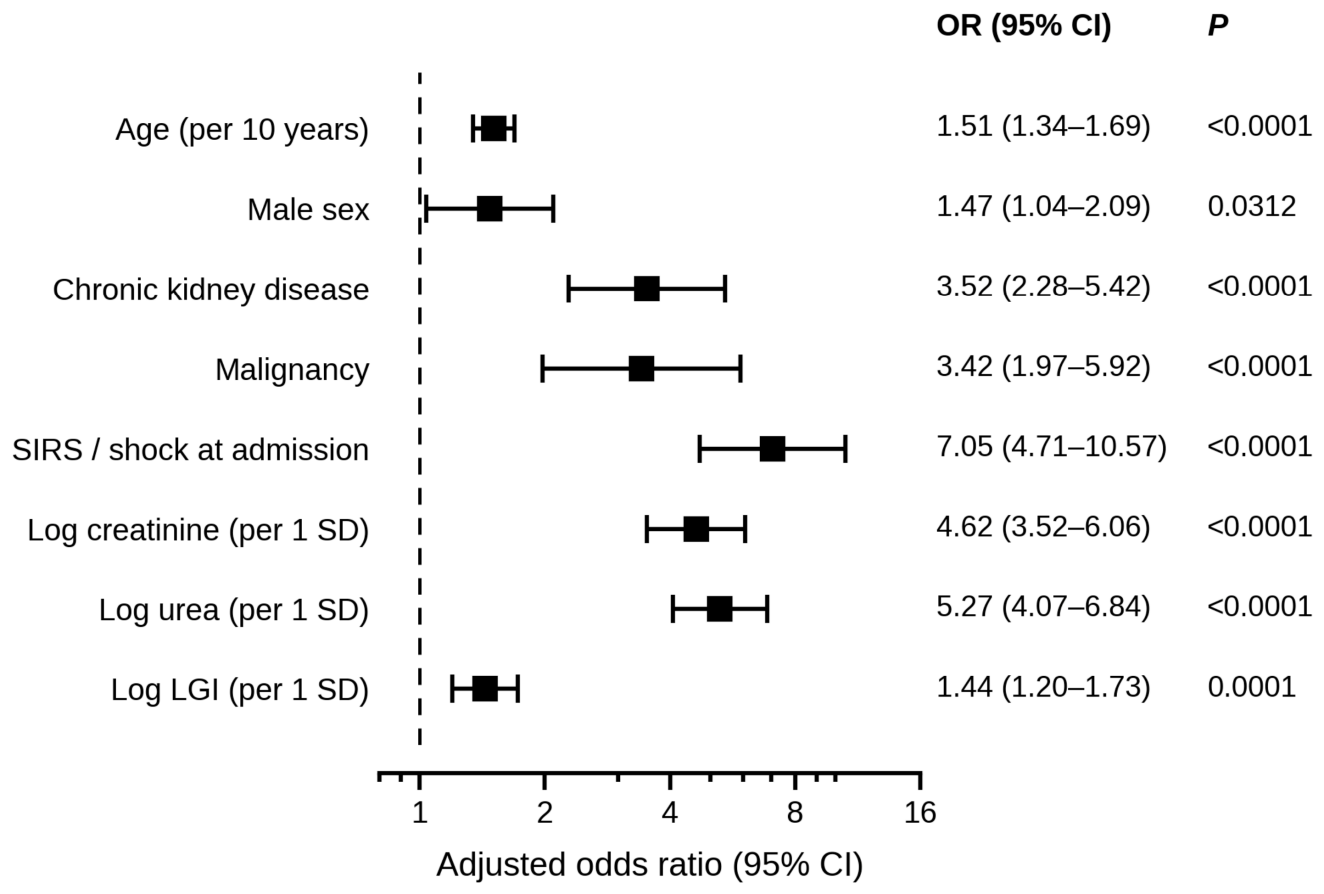
Variables independently associated with in-hospital mortality in multivariable logistic regression. Squares represent adjusted odds ratios and horizontal bars the corresponding 95% confidence intervals, plotted on a logarithmic scale; the vertical dashed line marks the null value (OR = 1). Continuous laboratory variables were natural log-transformed and are expressed per 1 standard deviation increment, and age is expressed per 10-year increment. CI, confidence interval; LGI, leuko-glycemic index; OR, odds ratio; SD, standard deviation; SIRS, systemic inflammatory response syndrome.

**Table 1:** Baseline characteristics and etiological distribution of survivors and non-survivors.

Variable	Group 1—Survivors (*n* = 1625)	Group 2—Non-Survivors (*n* = 302)	*p*
Age, median (IQR), years	61 (45–72)	73 (62–80)	<0.0001
Male sex, *n* (%)	886 (54.5%)	181 (59.9%)	0.08
**Etiology of acute surgical abdomen, n (%)**			*p* < 0.0001 *
Acute appendicitis	523 (32.2%)	28 (9.3%)	<0.0001
Bowel obstruction	395 (24.3%)	64 (21.2%)	0.27
Perforated viscus	204 (12.6%)	95 (31.5%)	<0.0001
Strangulated hernia	174 (10.7%)	36 (11.9%)	0.60
Mesenteric ischemia	62 (3.8%)	57 (18.9%)	<0.0001
Acute cholecystitis	101 (6.2%)	5 (1.7%)	0.002
Acute pancreatitis	65 (4.0%)	12 (4.0%)	1.0
Acute diverticulitis	62 (3.8%)	5 (1.7%)	0.09
Other/unspecified	39 (2.4%)	0 (0%)	0.01
**Comorbidities, *n* (%)**			
Diabetes mellitus	133 (8.2%)	27 (8.9%)	0.65
SIRS/shock at admission	195 (12.0%)	157 (52.0%)	<0.0001
Chronic kidney disease	114 (7.0%)	66 (21.9%)	<0.0001
Active malignancy	81 (5.0%)	48 (15.9%)	<0.0001
Hypertension	618 (38.0%)	166 (55.0%)	<0.0001
Ischemic heart disease	228 (14.0%)	85 (28.1%)	<0.0001

Continuous variables are presented as median (interquartile range, IQR 25th–75th percentile) and were compared using the Mann–Whitney U test. Categorical variables are presented as *n* (%) and were compared using the chi-square test. A two-tailed *p*-value of less than 0.05 was considered statistically significant. Sex was compared as a single binary variable (Pearson χ^2^). * The overall *p*-value for etiology refers to the χ^2^ test across all categories; category-specific *p*-values compare each etiology versus all others.

**Table 2:** Comparison of baseline biochemical parameters between the two study groups.

Parameters	Group 1—Survivors (*n* = 1625) Median [IQR25–75]	Group 2—Non-Survivors (*n* = 302) Median [IQR25–75]	*p* Value
Plasma glucose (mg/dL)	117 [100–143]	130 [101–168]	0.0004
Urea (mg/dL)	33.4 [25.5–48.4]	70.6 [44.2–110.2]	<0.0001
Creatinine (mg/dL)	0.85 [0.74–1.08]	1.43 [0.94–2.32]	<0.0001
AST (U/L)	22 [16–35]	31 [19–68]	<0.0001
ALT (U/L)	23 [15–44]	24 [13–45]	0.43

Values are presented as median [interquartile range, IQR 25–75]. AST, aspartate aminotransferase; ALT, alanine aminotransferase.

**Table 3:** Discriminative performance of NLR, MLR, PLR, and SII for predicting in-hospital mortality.

Index	AUC	AUC 95% CI	Cut-Off	Sensitivity %	Sens. 95% CI	Specificity %	Spec. 95% CI	LR−	LR+
NLR	0.597	0.558–0.636	7.545	58.28	52.65–63.70	57.05	54.63–59.43	0.73	1.36
MLR	0.580	0.541–0.618	0.585	57.28	51.65–62.74	58.77	56.36–61.14	0.73	1.39
PLR	0.593	0.554–0.632	206.7	59.93	54.32–65.30	59.38	56.98–61.75	0.68	1.48
SII	0.581	0.542–0.620	3290	36.4	31.18–41.97	80.7	78.71–82.55	0.79	1.89

**Table 4:** Multivariable logistic regression for in-hospital mortality.

Variable	β	SE	Adjusted OR	95% CI	*p*
SIRS/shock at admission	1.953	0.206	7.05	4.71–10.57	<0.0001
log urea (per 1 SD)	1.662	0.132	5.27	4.07–6.84	<0.0001
log creatinine (per 1 SD)	1.530	0.139	4.62	3.52–6.06	<0.0001
Chronic kidney disease	1.258	0.221	3.52	2.28–5.42	<0.0001
Active malignancy	1.230	0.281	3.42	1.97–5.92	<0.0001
Age (per 10 years)	0.412	0.059	1.51	1.34–1.69	<0.0001
Male sex	0.385	0.178	1.47	1.04–2.09	0.031
log LGI (per 1 SD)	0.365	0.093	1.44	1.20–1.73	0.0001

Model: *n* = 1927; 302 events; 38 events per variable. Discrimination AUC 0.765 (95% CI 0.74–0.79); calibration Hosmer–Lemeshow *p* = 0.70; Nagelkerke R^2^ = 0.25; all variance inflation factors < 1.7. Continuous laboratory variables were natural log-transformed and are expressed per 1 standard deviation increment. CI, confidence interval; LGI, leuko-glycemic index; OR, odds ratio; SD, standard deviation; SIRS, systemic inflammatory response syndrome.

## Data Availability

The data presented in this study are available on request from the corresponding author. The data are not publicly available due to privacy restrictions.

## Supplementary Materials

The following supporting information can be downloaded at: https://www.mdpi.com/article/10.3390/medicina62101879/s1, Table S1: STROBE Statement—Checklist of Items for Cohort Studies.

## Abbreviations

The following abbreviations are used in this manuscript:

LGI	Leuko-glycemic index
NLR	Neutrophil-to-lymphocyte ratio
PLR	Platelet-to-lymphocyte ratio
MLR	Monocyte-to-lymphocyte ratio
ROC	Receiver operating characteristic
AUC	Area under the curve
AST	Aspartate aminotransferase
ALT	Alanine aminotransferase
SIRS	Systemic Inflammatory Response Syndrome
SII	Systemic immune-inflammation index
MSOF	Multi-system Organ Failure
STEMI	ST-elevation myocardial infarction
COVID-19	Coronavirus Disease 2019
UCPU-SMURD	Clinical Emergency Unit—Mobile Emergency Service for Resuscitation and Extrication
WBC	White blood cells
AKI	Acute kidney injury
CABG	Coronary artery bypass grafting
