# Supplementary material for: Admission Leuko-Glycemic Index and In-Hospital Mortality in Patients with Acute Surgical Abdomen: An Exploratory Retrospective Cohort Study
Source: Medicina (Kaunas). 2026 Sep 28;62(10):1879. doi: 10.3390/medicina62101879 (PMC13707029; doi:10.3390/medicina62101879)
Supplement: Supplementary file 1 [file medicina-62-01879-s001.zip › medicina-4572155-supplementary.pdf]

Table S1: STROBE Statement — Checklist of Items for Cohort Studies.

Section	Item No	Recommendation	Reported on page/section	Manuscript text / location
Title and abstract	1a	Indicate the study's design with a commonly used term in the title or the abstract	Title; Abstract	Title states "a retrospective cohort study"; Abstract states "single-center retrospective observational study"
	1b	Provide in the abstract an informative and balanced summary of what was done and what was found	Abstract	Abstract now includes explicit a priori hypothesis ("We hypothesized that elevated admission LGI..."), methods, AUCs for all 5 indices, adjusted OR for LGI, and cautious conclusion
Introduction	2	Explain the scientific background and rationale for the investigation being reported	Introduction, paras 1–6	Expanded background comparing LGI's dual metabolic-inflammatory nature to purely cellular ratios (NLR/MLR/PLR/SII); literature gap explicitly stated
	3	State specific objectives, including any prespecified hypotheses	Introduction, final 2 paragraphs	New explicit hypothesis added: "We hypothesized that elevated admission LGI values would be independently associated with higher in-hospital mortality... and would show discriminative performance at least comparable to established inflammation-based indices"
Methods	4	Present key elements of study design early in the paper	2.1 Study population	Retrospective single-center cohort design stated at outset; operational case definition now added
	5	Describe the setting, locations, and relevant dates, including periods of recruitment, exposure, follow-up, and data collection	2.1 Study population	UCPU-SMURD Emergency Department, Târgu-Mureș, Romania; March 2019 – March 2021; pre-/post-COVID split quantified (54.03% vs. 45.97%)
	6a	Give the eligibility criteria, and the sources and methods of selection of participants; describe methods of follow-up	2.1 Study population	New operational definition of acute surgical abdomen added (sudden-onset pain <72h, peritoneal signs/radiological evidence, confirmed intraoperatively); exclusion criteria itemized: 148 missing data, 136 collection errors, 55 age<18, 68 transfers
	6b	For matched studies, give matching criteria and number of exposed/unexposed	Not applicable	Not a matched-cohort design
	7	Clearly define all outcomes,	2.3 Study outcome	Outcome: in-hospital mortality. Predictors: LGI, NLR, MLR, PLR, SII

		exposures, predictors, potential confounders, and effect modifiers; give diagnostic criteria if applicable		(formulas given). New: explicit diagnostic criteria added for SIRS (≥2 of temperature/HR/RR/leukocyte criteria) and shock (SBP<90 or MAP<65 + hypoperfusion/vasopressor need)
	8*	For each variable of interest, give sources of data and details of methods of assessment (measurement); describe comparability of assessment methods if more than one group	2.2 Data collection	Data extracted from ED charts, surgical observation charts, and Hipocrate hospital information system; identical protocol for both groups
	9	Describe any efforts to address potential sources of bias	2.1 Study population; Discussion	New: comparison of baseline age/sex/etiology between excluded (n=407) and included (n=1,927) patients showed no significant difference, addressing selection bias concern; pandemic case-mix and optimism bias also discussed
	10	Explain how the study size was arrived at	**2.1 Study population — RESOLVED**	New paragraph added verbatim: "As this was a retrospective observational study, no a priori sample size calculation was performed... representing a convenience sample determined by the observation window rather than by a statistical power calculation." This item, previously flagged by the reviewer, is now fully addressed
	11	Explain how quantitative variables were handled in the analyses; if applicable, describe groupings and why	2.4 Statistical analysis; 3.5	Non-normal continuous variables reported as median (IQR); LGI dichotomized at Youden-optimal cut-off (1,665); log-transformation used for regression variables
	12a	Describe all statistical methods, including those used to control for confounding	2.4 Statistical analysis	Mann-Whitney U; ROC/AUC with Wilson-Hybrid Score CIs; multivariable logistic regression with expanded covariate justification (clinical relevance + univariable p<0.10, explicit rationale for each retained covariate)
	12b	Describe any methods used to examine subgroups and interactions	Discussion, Limitations	Etiological subgroup analysis explicitly noted as not performed (acknowledged limitation); no formal interaction testing
	12c	Explain how missing data were addressed	2.1 Study population — EXPANDED	New: full breakdown of the 407 excluded patients by exact reason (148 missing data / 136 collection errors / 55 age<18 / 68 transfers),

				plus reassurance that excluded vs. included patients did not differ in age, sex, or etiology
	12d	If applicable, explain how loss to follow-up was addressed	Not applicable	In-hospital outcome only; no post-discharge attrition applicable
	12e	Describe any sensitivity analyses	**3.6 Multivariate logistic regression — NEW**	New paragraph added: diabetes mellitus prevalence reported (160, 8.3%; 133/8.2% Group 1 vs. 27/8.9% Group 2, p=0.65); sensitivity analysis adjusting for diabetes status (adjusted OR 1.43, 95% CI 1.18–1.72, p<0.0001) and analysis excluding diabetics (AUC 0.649) both now reported
Results	13a	Report numbers of individuals at each stage of study (eligible, examined, confirmed eligible, included, analysed)	2.1 Study population; Figure 1	2,334 screened → 407 excluded (itemized) → 1,927 included; flow diagram in Figure 1
	13b	Give reasons for non-participation at each stage	**2.1 Study population — RESOLVED**	Exact counts now given per exclusion reason (previously only categories were listed without counts)
	13c	Consider use of a flow diagram	Figure 1	Patient flow diagram included
	14a	Give characteristics of study participants (demographic, clinical, social) and information on exposures/potential confounders	3. Results opening; 3.1–3.3; Table 1	New: full etiological breakdown added (appendicitis 28.6%, bowel obstruction 23.8%, perforated viscus 15.5%, strangulated hernia 10.9%, mesenteric ischemia 6.2%, cholecystitis 5.5%, pancreatitis 4.0%, diverticulitis 3.5%, other 2.0%); age/sex/hematologic/biochemical comparisons by group
	14b	Indicate number of participants with missing data for each variable of interest	2.1 Study population (cohort-level only)	Missing-data exclusions reported at cohort level (148 patients); still not broken down per individual variable — residual gap
	14c	Summarise follow-up time (average and total amount)	Not applicable / brief	Follow-up restricted to index hospital admission (discharge-alive vs. in-hospital death)
	15*	Report numbers of outcome events or summary measures over time	3. Results, opening paragraph	1,625 (84.3%) discharged alive; 302 (15.7%) in-hospital deaths
	16a	Give unadjusted and confounder-adjusted estimates and their precision (95% CI); clarify which confounders were adjusted for and why	3.4–3.6; Figure 8	Unadjusted AUCs for all 5 indices; adjusted ORs with 95% CI for full model; new diabetes-adjusted sensitivity OR also reported with 95% CI
	16b	Report category	3.4–3.5	Youden-optimal cut-offs reported

		boundaries when continuous variables were categorized		per index (e.g., LGI 1,665; SII 3.29×10^3)
	16c	If relevant, consider translating estimates of relative risk into absolute risk for a meaningful time period	Not applicable	ORs reported; absolute-risk translation not performed (single-admission binary outcome)
	17	Report other analyses done (subgroups, interactions, sensitivity analyses)	**3.6 — RESOLVED**	Diabetes-status sensitivity analysis now integrated directly into Results (previously only in reviewer correspondence); etiological subgroup analysis still noted as not performed
Discussion	18	Summarise key results with reference to study objectives	4. Discussion, opening paragraphs	Key results summarized against the now-explicit prespecified hypothesis
	19	Discuss limitations of the study, taking into account sources of potential bias or imprecision; discuss direction and magnitude of any potential bias	4. Discussion, Limitations	Retrospective single-center design, cohort-derived cut-off (optimism bias), group imbalance, pandemic-period overlap, unmeasured confounders (corticosteroids, nutritional status; diabetes now addressed via sensitivity analysis)
	20	Give a cautious overall interpretation of results considering objectives, limitations, multiplicity of analyses, and other evidence	4. Discussion; 5. Conclusions	LGI framed as showing "modest" discrimination; explicitly not recommended as stand-alone tool
	21	Discuss the generalisability (external validity) of the study results	4. Discussion	Single-center/tertiary case-mix and pandemic-period overlap discussed; call for multicenter external validation
Other information	22	Give the source of funding and the role of the funders	Funding statement	"This research received no external funding."

**Starred items should be given separately for exposed and unexposed groups (here: survivors, Group 1, vs. non-survivors, Group 2).*
Source: Checklist adapted from the STROBE Statement (von Elm et al., 2007), cohort study version (strobe-statement.org).
